# Gender Differences in Correlates of Loneliness among Community-Dwelling Older Koreans

**DOI:** 10.3390/ijerph19127334

**Published:** 2022-06-15

**Authors:** Young Bum Kim, Seung Hee Lee

**Affiliations:** 1 Institute of Aging, Hallym University, Chuncheon 24252, Korea; twoponej@gmail.com; 2 Department of Nursing, The University of Ulsan, Ulsan 44610, Korea

**Keywords:** loneliness, social network, older adults, Korea

## Abstract

Background: Despite a relatively large number of studies exploring late-life loneliness, few studies have compared gender differences in the correlates of loneliness of older adults. Thus, we examined the gender differences in correlates of loneliness among community-dwelling older adults. Methods: This study was a secondary analysis of data from a parent study conducted among community-dwelling Koreans 65 years of age or older. Loneliness was measured by the 20-item Revised University of California Los Angeles Loneliness Scale. As potential correlates, demographic, health-related, and social variables were included. Multivariate hierarchical regression analyses were performed separately by gender. Results: Men were more likely to be lonely than women, after controlling for demographic, health-related, and social variables. A social network of family ties and being married were found to be inversely correlated with loneliness in men but not in women. A social network of friendship ties and participation in a variety of community activities were inversely correlated with loneliness in both men and women. Conclusions: A social network of family ties and being married may help reduce late-life loneliness, particularly among men. This study highlights the importance of considering gender differences in the design of strategies for preventing and alleviating late-life loneliness.

## 1. Introduction

Old age is characterized by life transitions that increase the risk of loneliness, such as loss of social roles through retirement; deaths of spouse, relatives, and friends; and deteriorating health condition [1,2]. The prevalence of loneliness in older adults has been estimated to vary from 21% to 55% [3,4]. Enduring loneliness has long been recognized as a significant predictor of various health problems of older adults, such as increased risk of depressive symptoms, cognitive decline, functional disability, and mortality [5,6,7].

Loneliness in the older adults has been reported to be associated with demographic variables (e.g., gender, low socioeconomic status, and education) and health-related variables (e.g., functional status, self-rated health, and depressive symptoms) [8,9]. Education is known to play a protective role in loneliness [8,10]. Education may provide individuals with a variety of resources that can help them cope with loneliness [10]. Poverty or financial strain decreases psychological well-being and leads to a higher risk of developing loneliness [4,8]. Functional disability and poor self-rated health, which impede social engagement and lead to loneliness, also increase with age and tend to be more common for women than men [7,8,9]. Many studies have shown that the prevalence of loneliness is higher in older women compared with older men [4,7,8,10]. Some possible reasons for this may be differences relating to gender in demographic and health variables that predict loneliness in older adults. Older women are more likely to be poorer or have a lower educational attainment than older men [7,10]. Older women’s health status is also usually worse than that of older men [7,10], and a poor health status imposes limitations on their social interactions [11], making older women more vulnerable to loneliness than older men. In addition, women tend to live longer than men and are, therefore, at increased risk for loneliness after their husband’s death [10,12]. However, the effect of marital status on loneliness has been reported to be greater among older men than older women because men are intrinsically reliant on their spouses for intimacy and support [13]. In general, older men receive more emotional gratification from their spouses and rely more heavily on their spouses for social connections [13,14]. Because older men tend to emotionally benefit more from marriage than do older women, older men have more to lose from their spouse’s death [14]. As a result, the absence of a spouse has more adverse effects on men than on women. Most studies in Western populations have shown that the loss of a spouse produces a stronger negative effect on psychological well-being (e.g., depressive symptoms, loneliness) in men than in women [13,14].

Engagement in various community activities can reduce loneliness in older adults by providing human contact and intimacy [8,9,15]. The gerontological literature suggests that social networks and social connections can be important for psychological well-being in later life [11,16]. Having a strong and supportive social network has been linked to improved mental and physical health of older adults [8,11,13]. The size and diversity of one’s social network have also been associated inversely with loneliness [8,11,17]. However, these effects of social networks on physical and mental health can be gender specific. For example, a longitudinal study in Germany found that a stronger social network was related to decreased physical health-related quality of life in older men, but not in older women [18]. Women are usually expected to play the role of caregiver, whereas men are not [19]. Thus, older men having health-related issues gain more benefits from social networks, such as their family’s caregiving, and instrumental and emotional support, than do older women [20,21]. In the same study, the size of the social network was reported not to be linked to the physical health-related quality of life in German older women [18]. Other studies in Western populations also found that having a greater connection to social networks was inversely related to depressive symptoms in men, but not in women [13]. This may be because, compared to men, women tend to be more affected by the quality but not the quantity of social networks [22]. Women are more prone to experiencing greater support from small and closer social networks [23]. By comparison, a study in Korean older adults showed that social network variables (e.g., number of close friends, and the social activities) were significantly linked to cognitive function in women, but not in men [24]. Another study in European older adults suggested that men benefited more in terms of quality of life from social networks and social support, whereas women benefited more from social participation, such as participation in a group or community [25]. These gender differences observed in the association between social networks and physical or mental health may be due to the gendered nature of social networks. In the nature of their interactions with social networks, men and women may differ [26]. Older men are less active in building new social ties and participating in social activities compared with older women [26,27]. Older men tend to prefer more acquainted ties, whereas older women tend to have more extensive social ties by creating new close ties [26]. Although gender-specific effects of social networks on health have been extensively studied in older adults, the findings are inconsistent [13,18,24]. Additionally, we found very few studies regarding gender differences in the association between social networks and loneliness [13].

Scholars have noted the importance of considering gender differences when exploring correlates or risk factors of loneliness among older adults [12,28]. However, although many studies have examined the correlates of loneliness in older age, few have investigated gender differences [9,29]. Furthermore, most studies on this topic have been conducted in Western countries [12,13,28]; studies in Asian societies are relatively sparse. To our knowledge, no study has examined the gender differences in the risk factors that predict loneliness among Korean older adults. Therefore, to fill this gap in the knowledge, the present study examined gender differences in correlates of loneliness among community-dwelling older Koreans.

## 2. Materials and Methods

### 2.1. Study Design and Participants

The present study was a secondary examination of the data from a larger cross-sectional survey on aging of community-dwelling individuals aged 65 years old or older, in Chuncheon city, which is located near Seoul, between September and November 2017. Demographic, health, and social data were collected by a structured questionnaire. For data collection, quota sampling was employed to balance for sex, age, and residential area. The quotas were determined according to the sample distributions of sex (men or women), age (65–69, 70s, 80s), and residential area (rural or urban). Participants were informed about the study and invited to participate by a proficient interviewer at churches, community health posts, senior citizen clubs, and senior centers. A total of 1000 older adults participated in the study. The details of data collection and the study design of the parent study were reported previously [16].

### 2.2. Measures

We employed instruments measuring demographic, health-related, and social variables in the current study. The demographic variables included the following: gender, age, education, marital status, household income, job, and residential area. Household income was classified as follows: low (fulfilled the criteria for absolute poverty status); moderate (received the basic old-age pension); high (the others). Health-related variables included the following: self-rated health, number of chronic diseases, depressive symptom, and instrumental activities of daily living (IADL). The 10-item Korean IADL Scale was used for assessing the ability to independently perform 10 activities, including household chores, going out a short distance, and personal grooming. This measure has been reported to have adequate reliability [30]. Subjects who were unable to perform one or more activities were coded to be functionally dependent in IADL. Depressive symptoms were assessed with the 15-item Geriatric Depression Scale—Short Form (GDS-S). Higher scores show an increase in depressive symptoms, with a possible range from 0 to 15. The reliability and validity of this scale were found to be adequate in previous research [31,32]. The alpha reliability of the GDS-S in this study was 0.88.

Social variables included the following: living alone, number of people living in respondent’s household, participation in a variety of community activities, and social network. Participation in a variety of community activities was measured by the number of participations among 6 categories of church or other religious groups, community houses for seniors, leisure groups, art or music groups, alumni societies or societies for people from the same hometown, volunteer groups, and interest groups. Respondents rated each of the activities according to how often they participate. The item responses are ‘never (1)’ to ‘every day (10)’. Total scores ranged from 6 to 60.

Social network participation was assessed by the Lubben Social Network Scale—Revised (LSNS-R) [33]. This scale measures information related to the extent of the individual’s active social networks (e.g., number of friends and family/kin seen or heard from ≥ one times/month), frequency of social contact with friends and family/kin, perceived support network (e.g., friends and family/kin who could be asked for help), and perceived confidant network (e.g., friends and family/kin to whom the individual could speak about private affairs). The measure consists of a set of six questions that rate family ties and a corresponding set of six questions that rate friendship ties. Scores for each subscale for social networks of family ties and friendship ties were summed. Higher scores show greater social networks, with a possible range from 0 to 30. The reliability and validity of the LSNS-R were found to be adequate in existing research [34,35]. The alpha reliability of the LSNS-R was 0.90 in the present study.

Loneliness was assessed by the 20-item Revised University of California Los Angeles Loneliness Scale (UCLA LS-R), ref. [36], which was validated in Korean samples [37]. The scale comprises of 20 statements ranked by subjects (1 = I rarely feel like this, 4 = I frequently feel like this), with a possible range from 20 to 80. A higher score indicates that the individual experiences greater levels of loneliness. The reliability and validity of the scale were found to be adequate [36,37]. In this study, the alpha coefficient was 0.89 for UCLA LS-R.

### 2.3. Ethical Considerations

This research was approved by the Hallym Institutional Review Board (HIRB-2016-008-3-RCC). Written consent was given by all subjects. The present study was carried out in compliance with the guidelines of the Declaration of Helsinki.

### 2.4. Statistical Analysis

Descriptive analyses were conducted to characterize the participants. T-tests and the chi-square test were used to examine the differences of variables between men and women. To evaluate which explanatory variables predict loneliness for men and women, we performed multivariate hierarchical regression analyses. In the first block, demographic variables were introduced. In the second block, health-related variables of self-rated health, number of chronic diseases, dependence in IADL, and depressive symptoms were included. Finally, in the third block, social variables were entered: number of people living in the participant’s household, social network of family ties, social network of friendship ties, and participation in a variety of community activities. Analyses were carried out in STATA, version 15.1, and were two-tailed with an alpha level of 0.05.

## 3. Results

### 3.1. Descriptive Analyses

Table 1 presents the distribution of study variables by gender. Of all the participants, 58.6% were women and 41.4% were men. There were significant differences between men and women in terms of age, marital status, education, household income, having a job, number of chronic diseases, IADL, self-rated health, depressive symptoms, living alone, number of people living in one’s household, and loneliness. Compared to men, women were likely to be older, unmarried, more depressed, living alone, have a lower education and household income, and have poor self-rated health. Additionally, more women than men were likely to report loneliness (women:33.97 versus men:32.40; *p* = 0.018). However, no gender differences were found for social network of family ties, social network of friendship ties, and participation in a variety of community activities. 

### 3.2. Multivariate Hierarchical Regression Analyses

The multivariate hierarchical regression analyses for the total sample indicated that men were more likely to be lonely than women, after adjusting for demographic, health-related, and social variables (Table 2).

Table 3 shows the results of multivariate hierarchical regression analyses for loneliness of older men. The final model explained 72% of the total variance associated with loneliness. In step 1, the demographic variables age, being married, education, household income, living in an urban area, and having a job explained 30% of the variance. Step 2 explained an additional 35% with the health-related variables self-rated health, number of chronic diseases, being dependent in IADL, and depressive symptoms. The social variables number of people living in the participant’s household, social network of family ties, social network of friendship ties, and participation in a variety of community activities explained a further 7% of the variance. The significant predictors in the final model for loneliness of older men were depressive symptom (β = 0.57), social network of friendship ties (β = −0.19), social network of family ties (β = −0.15), participation in a variety of community activities (β = −0.10), married (β = −0.09), education (β = −0.07), and living in urban areas (β = −0.06).

Table 4 shows the results of multivariate hierarchical regression analyses for loneliness of older women. The final model explained 61% of the total variance associated with loneliness. In step 1, the demographic variables age, being married, education, household income, living in urban areas, and having a job explained 19% of the variance. Step 2 explained an additional 35% with the health-related variables self-rated health, number of chronic diseases, dependent in IADL, and depressive symptoms. The social variables number of people living in the participant’s household, social network of family ties, social network of friendship ties, and participation in a variety of community activities explained a further 7% of the variance. The significant predictors in the final model for loneliness of older women were depressive symptoms (β = 0.51), social network of friendship ties (β = −0.24), participation in a variety of community activities (β = −0.09), and having a job (β = −0.08).

## 4. Discussion

In this study, we investigated the gender differences in correlates of loneliness using a sample of community-dwelling, older Koreans. Results from our model predicting loneliness revealed that depressive symptoms were most strongly correlated with loneliness in both men and women. This was in line with evidence that depressive symptoms were highly related to loneliness in older adults [8,38]. Existing studies have reported that loneliness and depressive symptoms frequently co-occur, and there can be reciprocal influences over time between them [39]. Due to the cross-sectional nature of our study, it is impossible to examine whether depressive symptoms activate loneliness or whether loneliness activates depressive symptoms. Further research is warranted to clarify the directionality of the relationship. In this study, among both men and women, older adults who participated more frequently in a variety of community activities had a lower risk of loneliness than those who did not. Engagement in diverse social activities in later life has been reported to provide opportunities for older adults to exchange similar interests, share life experiences, and facilitate social connections, which may alleviate their loneliness [40,41]. Our findings support previous literature reporting the beneficial effect of social activity on late-life loneliness in both men and women [15,41]. Being married was associated with a lower likelihood of loneliness, but only among men, which is consistent with a Japanese study that showed the increased loneliness risk associated with the absence of a spouse was stronger for men than for women [42].

Although the extent of a person’s social networks has been considered to be a predictor of loneliness, very little is known about which aspects of the social network are salient to late-life loneliness [8,16]. In the current study, using the LSNS-R, we divided the social network into two sub-dimensions: social network of family ties and social network of friendship ties [33]. In our analyses, among both men and women, social network of friendship ties was the second strongest predictor of loneliness. Older men and women having a greater level of social networks of friendship ties had lower risk of loneliness than those who did not. However, interestingly, having a social network of family ties was found to be inversely correlated with loneliness in men but not in women. The cause for this finding is unclear, but a possible interpretation is that men and women differ in the nature of their social relations. For example, older men prefer more familial and well-known social networks, whereas older women tend to enlarge their social networks via the creation of new close ties [26]. Studies have demonstrated that men perceive more positive marital exchanges than women [43]. Compared to women, men are more inclined to rely on spousal relationships for maintaining their physical and mental well-being in late life, whereas women rely more on extended network members, including close friends or neighborhood, thereby diversifying the potential types of support available to women [7,26]. In our study, we found that, after adjusting for potential confounders, men had a higher risk of loneliness than women in the total study sample. This result is somewhat unexpected, because the existing literature reports that older women have higher odds of loneliness than older men. However, a study on loneliness among Japanese older adults also found that men were more likely to be lonely than were women after controlling for covariables [42]. Potential explanations for this may be that family and spouse factors are more strongly associated with loneliness for older men than for older women in Asian culture [10,42]. Furthermore, women tend to live longer than men. Living without a spouse can shrink a women’s family networks, which may then reduce the impact of family networks on loneliness among women [44,45]. In our analyses, the contribution to the variance of health variables was equally strong for loneliness in both men and women, and was around 35%. However, the contribution to the variance in demographic variables was larger for men than for women (men, 30%; women, 19%). Education was inversely associated with loneliness in men but not in women. One reason for this may be the overall low level of education of older women in Korea [46]. In this sample, the levels of education of women were relatively low and homogenous, and are thus unlikely to hold much explanatory power. 

The implication of these findings is that there are important gender differences in the factors associated with loneliness at older ages and each gender may require different intervention strategies in alleviating loneliness. We should extend our knowledge on gender differences in loneliness and its predictors for the design of more targeted interventions to prevent and reduce loneliness. This study suggests that having a greater social network of friendship ties is also an important protective factor against late-life loneliness in both men and women. Being married and having a social network of family ties are central to late-life loneliness, particularly among men. For older men, good marital quality and intergenerational family relationships may be important to late-life loneliness.

There are several limitations to the current study. First, due to the cross-sectional design of this study, the causal relations between loneliness and its identified correlates cannot be determined. More research using longitudinal data is needed to explore the causal and temporal relationships between loneliness and its correlates in later life. Second, our findings are based on a global measure of loneliness. We did not distinguish between dimensions of loneliness, such as emotional and social loneliness [9]. Thus, we cannot know which subtypes of loneliness are specifically associated with different risk factors. Further research using different dimensions of loneliness will be able to demonstrate the relationships between specific domains of loneliness and their correlates. Third, since this study was conducted in one geographic area of Korea, the generalizability of the findings may be limited. Fourth, this study was limited to self-reported data, and did not include more objective data, such as physicians’ diagnoses or income data. Self-reports may be subject to errors of recall or reporting. Finally, studies have shown that the quality rather than the quantity of social networks may be more important for predicting psychological health in late life [47]. However, our scale did not evaluate the qualitative nature of social networks. More research using integrated measures including both qualitative and quantitative aspects of social networks is warranted. Despite these limitations, this study adds a unique finding to the current literature by examining gender differences in correlates of late-life loneliness among non-Western populations. This study also suggests the importance of considering gender differences when designing strategies for preventing and alleviating late-life loneliness.

## 5. Conclusions

This community-based study showed that having a social network of family ties and being married may help reduce late-life loneliness, particularly among men. A social network of friendship ties and participation in a variety of community activities can play essential roles in decreasing late-life loneliness in both men and women. More research in diverse older populations, using cross-cultural analyses, is warranted to confirm the present findings.

## Figures and Tables

**Table 1:** Descriptive characteristics of the participants by gender (*n* = 1000).

	Mean ± SD or %			
Variable	Men (*n* = 414)	Women (*n* = 586)	t or X^2^	*p*-Value
**Demographic variables**				
Age (year)	74.36(6.11)	75.30(6.53)	2.29	0.022
Marital status			180.93	0.000
Unmarried, divorced or widowed	77(18.6)	360(61.4)		
Married	337(81.4)	226(38.6)		
Education (year)	9.58(4.21)	5.51(3.95)	−15.59	0.000
Household income			58.46	0.000
Low	82(19.8)	248(42.3)		
Moderate	220(53.1)	244(41.6)		
High	112(27.2)	94(16.1)		
Residential area			1.04	0.307
Rural	118(28.5)	150(25.6)		
Urban	296(71.5)	436(74.4)		
Having a job			4.30	0.038
No	252(60.9)	394(67.2)		
Yes	162(39.1)	192(37.8)		
**Health related variables**				
Number of chronic diseases	1.36(1.06)	1.66(1.21)	4.04	0.000
Instrumental activity of daily living			194.96	0.000
Independent	146(35.3)	463(79.0)		
Dependent	268(64.7)	123(21.0)		
Self-rated health	2.53(1.01)	2.95(1.02)	6.29	0.000
Depressive symptom	2.00(3.64)	2.89(4.01)	3.60	0.000
**Social variables**				
Living alone	65(15.70)	240(40.96)	73.00	0.000
Number of people living in participant ’s household	1.13(.92)	0.92(1.17)	−3.06	0.002
Social network of family ties	17.89(5.59)	18.10(5.46)	0.61	0.544
Social network of friendship ties	17.92(6.15)	17.23(5.98)	−1.76	0.078
Participation in a variety of community activities	17.61(6.23)	17.90(5.87)	0.73	0.465
**Loneliness**	32.40(10.83)	33.97(9.89)	2.38	0.018

**Table 2:** Multivariate hierarchical regression model predicting loneliness for total sample.

Variable	Model 1		Model 2		Model 3	
	Coefficients (SE)	β	Coefficients (SE)	β	Coefficients (SE)	β
Men	2.72(0.74)	0.13 ***	2.28(0.63)	0.11 ***	1.50(0.58)	0.07 *
Age	0.10(0.06)	0.06	−0.03(0.04)	−0.02	−0.05(0.04)	−0.03
Married	−1.93(0.79)	−0.09 *	−1.58(0.56)	−0.08 **	−1.40(0.52)	−0.07 **
Education (year)	−0.49(0.09)	−0.21 ***	−0.20(0.06)	−0.09 ***	−0.14(0.06)	−0.06 *
Moderate and high household income	−5.09(0.76)	−0.23 ***	−0.55(0.58)	−0.03	−0.58(0.57)	−0.03
Living in urban areas	−0.86(0.66)	−0.04	−0.87(0.47)	−0.04	−1.19(0.42)	−0.05 **
Having a job	−4.25(0.62)	−0.20 ***	−0.75(0.48)	−0.03	−1.04(0.44)	−0.05 *
Self-rated health			1.42(0.31)	0.14 ***	0.07(0.30)	−0.02
Number of chronic diseases			0.17(0.22)	0.43	0.35(0.21)	−0.04
IADL dependent			−0.37(0.57)	−0.02	−0.27(0.51)	−0.01
Depressive symptom			1.61(0.09)	0.61 ***	1.45(0.08)	0.55 ***
Number of people living in participant’s household					0.05(0.18)	0.00
Social network of family ties					−0.13(0.05)	−0.07 **
Social network of friendship ties					−0.38(0.05)	−0.23 ***
Participation in a variety of community activities					−0.17(0.04)	−0.10 ***
R^2^ change (adjusted)		0.22		0.35		0.08

Note. In household income, the low household income group was left out of the analysis as a reference group. Abbreviations: SE = standard errors; IADL = instrumental activity of daily living. * *p* < 0.05, ** *p* < 0.01, *** *p* < 0.001.

**Table 3:** Multivariate hierarchical regression model predicting loneliness for men.

Variable	Model 1		Model 2		Model 3	
	Coefficients (SE)	β	Coefficients (SE)	β	Coefficients (SE)	β
Age	0.17(0.09)	0.09	0.03(0.06)	0.02	0.06(0.06)	0.03
Married	−5.61(1.63)	−0.20 **	−2.45(1.09)	−0.09 *	−2.45(1.03)	−0.09 *
Education (year)	−0.48(0.11)	−0.19 ***	−0.30(0.08)	−0.12 ***	−0.19(0.07)	−0.07 *
Moderate and high household income	−7.30(1.49)	−0.27 ***	−1.79(1.11)	−0.07 *	−1.32(1.03)	−0.05
Living in urban areas	−1.96(0.95)	−0.08 *	−0.71(0.71)	−0.03	−1.36(0.63)	−0.06 *
Having a job	−2.87(0.92)	−0.13 **	0.47(0.76)	0.02	0.36(0.67)	0.02
Self-rated health			1.03(0.45)	0.10 *	−0.09(0.43)	−0.01
Number of chronic diseases			0.36(0.63)	0.02	0.53(0.56)	0.03
IADL dependent			−0.32(0.78)	−0.01	−0.44(0.70)	−0.02
Depressive symptom			1.90(0.15)	0.64 ***	1.69(0.14)	0.57 ***
Number of people living in participant’s household					0.54(0.28)	0.05
Social network of family ties					−0.22(0.07)	−0.11 **
Social network of friendship ties					−0.33(0.06)	−0.19 ***
Participation in a variety of community activities					−0.17(0.05)	−0.10 **
R^2^ change (adjusted)	0.30		0.35		0.07	

Note. In household income, the low household income group was left out of the analysis as a reference group. Abbreviations: SE = standard errors; IADL = instrumental activity of daily living. * *p* < 0.05, ** *p* < 0.01, *** *p* < 0.001.

**Table 4:** Multivariate hierarchical regression model predicting loneliness for women.

Variable	Model 1		Model 2		Model 3	
	Coefficients (SE)	β	Coefficients (SE)	β	Coefficients (SE)	β
Age	0.09(0.07)	0.06	−0.07(0.06)	−0.04	−0.11(0.06)	−0.07
Married	−0.23(0.85)	−0.01	−1.11(0.66)	−0.05	−0.88(0.61)	−0.04
Education (year)	−0.50(0.12)	−0.20 ***	−0.10(0.09)	−0.04	−0.12(0.09)	−0.05
Moderate and high household income	−4.22(0.87)	−0.21 ***	−0.44(0.68)	−0.02	−0.42(0.68)	−0.02
Living in urban areas	−0.02(0.88)	0.00	0.70(0.61)	−0.03	0.85(0.56)	−0.04
Having a job	−4.94(0.81)	−0.23 ***	−1.23(0.61)	−0.06 *	−1.73(0.56)	−0.08 **
Self-rated health			1.63(0.41)	0.17 ***	0.34(0.40)	0.03
Number of chronic diseases			−0.23(0.55)	−0.01	0.41(0.49)	0.03
IADL dependent			1.51(0.93)	0.06	1.31(0.86)	0.05
Depressive symptom			1.39(0.11)	0.56 ***	1.27(0.11)	0.51 ***
Number of people living in participant’s household					−0.19(0.23)	−0.02
Social network of family ties					−0.09(0.06)	−0.05
Social network of friendship ties					−0.40(0.06)	−0.24 ***
Participation in a variety of community activities					−0.15(0.06)	−0.09 *
R^2^ change (adjusted)	0.19		0.35		0.07	

Note. In household income, the low household income group was left out of the analysis as a reference group. Abbreviations: SE = standard errors; IADL = instrumental activity of daily living. * *p* < 0.05, ** *p* < 0.01, *** *p* < 0.001.

## Data Availability

The data presented in this study are available on request from the corresponding author. The data are not publicly available due to privacy.

## References

[1] Cohen-Mansfield J., Hazan H., Lerman Y., Shalom V. (2016). Correlates and predictors of loneliness in older adults: A review of quantitative results informed by qualitative insights. Int. Psychogeriatr..

[2] Victor C.R., Yang K. (2012). The prevalence of loneliness among adults: A case study of the United Kingdom. J. Psychol..

[3] Jamieson H.A., Gibson H.M., Abey-Nesbit R., Ahuriri-Driscoll A., Keeling S., Schluter P.J. (2018). Profile of ethnicity, living arrangements and loneliness amongst older adults in Aotearoa New Zealand: A national cross-sectional study. Australas. J. Ageing.

[4] Hansen T., Slagsvold B. (2016). Late-life loneliness in 11 European countries: Results from the generations and gender survey. Soc. Indic. Res..

[5] O’ Súilleabháin P.S., Gallagher S., Steptoe A. (2019). Loneliness, living alone, and all-cause mortality: The role of emotional and social loneliness in the elderly during 19 years of follow-up. Psychosom. Med..

[6] Montoliu T., Hidalgo V., Salvador A. (2019). The relationship between loneliness and cognition in healthy older men and women: The role of cortisol. Psychoneuroendocrinology.

[7] Guo L., An L., Luo F., Yu B. (2021). Social isolation, loneliness and functional disability in Chinese older women and men: A longitudinal study. Age Ageing.

[8] Dahlberg L., McKee K.J., Frank A., Naseer M. (2022). A systematic review of longitudinal risk factors for loneliness in older adults. Aging Ment. Health.

[9] Wolfers M.E.G., Stam B.E., Machielse A. (2022). Correlates of emotional and social loneliness among community dwelling older adults in Rotterdam, the Netherlands. Aging Ment. Health.

[10] Yang F. (2021). Widowhood and loneliness among Chinese older adults: The role of education and gender. Aging Ment. Health.

[11] Fiori K.L., Smith J., Antonucci T.C. (2007). Social network types among older adults: A multidimensional approach. J. Gerontol. B Psychol. Sci. Soc. Sci..

[12] Barreto M., Victor T., Hammond C., Eccles A., Richins M.T., Qualter P. (2021). Loneliness around the world: Age, gender, and cultural differences in loneliness. Pers. Individ. Dif..

[13] Santini Z.I., Fiori K.L., Feeney J., Tyrovolas S., Haro J.M., Koyanagi A. (2016). Social relationships, loneliness, and mental health among older men and women in Ireland: A prospective community-based study. J. Affect. Disord..

[14] Lee G.R., DeMaris A., Bavin S., Sullivan R. (2001). Gender differences in the depressive effect of widowhood in later life. J. Gerontol. B Psychol. Sci. Soc. Sci..

[15] McDaid D., Park A. (2021). Modelling the economic impact of reducing loneliness in community dwelling older people in England. Int. J. Environ. Res. Public Health.

[16] Kim Y.B., Lee S.H. (2019). Social support network types and depressive symptoms among community-dwelling older adults in South Korea. Asia Pac. J. Public Health.

[17] Gyasi R.M., Phillips D.R., Asante F., Boateng S. (2021). Physical activity and predictors of loneliness in community-dwelling older adults: The role of social connectedness. Geriatr. Nurs..

[18] Boehlen F.H., Maatouk I., Friederich H., Schoettker B., Brenner H., Wild B. (2021). Loneliness as a gender-specific predictor of physical and mental health-related quality of life in older adults. Qual. Life Res..

[19] Chipperfield J.G., Havens B. (2001). Gender differences in the relationship between marital status transitions and life satisfaction in later life. J. Gerontol. B Psychol. Sci. Soc. Sci..

[20] Liu H., Umberson D., Xu M. (2020). Widowhood and mortality: Gender, race/ethnicity, and the role of economic resources. Ann. Epidemiol..

[21] Caetano S.C., Silva C.M., Vettore M.V. (2013). Gender differences in the association of perceived social support and social network with self-rated health status among older adults: A population-based study in Brazil. BMC Geriatr..

[22] Orth-Gomer K. (2009). Are social relations less health protective in women than in men? Social relations, gender, and cardiovascular health. J. Soc. Pers. Relat..

[23] Haines V.A., Beggs J.J., Hurlbert J.S. (2008). Contextualizing health outcomes: Do effects of network structure differ for women and men?. Sex Roles.

[24] Lee S., Lee E., Youm Y., Cho H.S., Kim W.J. (2020). Gender differences in social network of cognitive function among community-dwelling older adults. Geriatr. Gerontol. Int..

[25] Tobiasz-Adamczyk B., Galas A., Zawisza K., Chatterji S., Haro J.M., Ayuso-Mateos J.L., Koskinen S., Leonardi M.J. (2017). Gender-related differences in the multi-pathway effect of social determinants on quality of life in older age—The COURAGE in Europe project. Qual. Life Res..

[26] Schwartz E., Litwin H. (2018). Social network changes among older Europeans: The role of gender. Eur. J. Ageing.

[27] Cornwell B. (2011). Independence through social networks: Bridging potential among older women and men. J. Gerontol. B Psychol. Sci Soc. Sci..

[28] von Soest T., Luhmann M., Hansen T., Gerstorf D. (2020). Development of loneliness in midlife and old age: Its nature and correlates. J. Pers. Soc. Psychol..

[29] Grover S., Verma M., Singh T., Dahiya N., Nehra R. (2019). Loneliness and its correlates amongst elderly attending non-communicable disease rural clinic attached to a tertiary care centre of North India. Asian J. Psychiatr..

[30] Park B., Jun J.K., Park J. (2014). Cognitive impairment and depression in the early 60s: Which is more problematic in terms of instrumental activities of daily living?. Geriatr. Gerontol. Int..

[31] Sheikh J.I., Yesavage J.A. (1986). Geriatric depression scale (GDS) recent evidence and development of a shorter version. Clin. Gerontol..

[32] Park N.S., Jang Y., Lee B.S., Chiriboga D.A., Chang S., Kim S.Y. (2018). Associations of a social network typology with physical and mental health risks among older adults in South Korea. Aging Ment. Health.

[33] Lubben J.E. (1988). Assessing social networks among elderly populations. Fam. Community Health.

[34] Crooks V.C., Lubben J.E., Petitti D.B., Little D., Chiu V. (2008). Social network, cognitive function, and dementia incidence among elderly women. Am. J. Public Health.

[35] Lubben J.E., Blozik E., Gillmann G., Iliffe S., von Renteln Kruse W., Beck J.C., Stuck A.C. (2006). Performance of an abbreviated version of the Lubben Social Network Scale among three European Community-dwelling older adult populations. Gerontologist.

[36] Russell D., Peplau L.A., Cutrona C.E. (1980). The revised UCLA loneliness scale: Concurrent and discriminant validity evidence. J. Pers. Soc. Psychol..

[37] Kim O. (1999). Predictors of loneliness in elderly Korean immigrant women living in the United States of America. J. Adv. Nurs..

[38] Golden J., Conroy R.M., Bruce I., Denihan A., Greene E., Kirby M., Lawlor B.A. (2009). Loneliness, social support networks, mood and wellbeing in community-dwelling elderly. Int. J. Geriatr. Psychiatry.

[39] Luanaigh C.O., Lawlor B.A. (2008). Loneliness and the health of older people. Int. J. Geriatr. Psychiatry.

[40] Bai Z., Wang Z., Shao T., Qin X., Hu Z. (2021). Association between social capital and loneliness among older adults: A cross-sectional study in Anhui Province, China. BMC Geriatr..

[41] Taylor H.O. (2020). Social isolation’s influence on loneliness among older adults. Clin. Soc. Work J..

[42] van den Broek T. (2017). Gender differences in the correlates of loneliness among Japanese persons aged 50–70. Australas. J. Ageing.

[43] Chan A., Malhotra C., Malhotra R., Ostbye T. (2011). Living arrangements, social networks and depressive symptoms among older men and women in Singapore. Int. J. Geriatr. Psychiatry.

[44] Harling G., Morris K.A., Manderson L., Perkins J.M., Berkman L.F. (2020). Age and gender differences in social network composition and social support among older rural South Africans: Findings from the HAALSI study. J. Gerontol. B Psychol. Sci. Soc. Sci..

[45] Powell V.D., Abedini N.C., Galecki A.T., Kabeto M., Kumar N., Silveira M.J. (2021). Unwelcome companions: Loneliness associates with the cluster of pain, fatigue, and depression in older adults. Gerontol. Geriatr. Med..

[46] Kim Y.B., Lee S.H. (2015). Prevalence and risk factors associated with prehypertension by gender and age in a Korean population in the KNHANES 2010–2012. Iran. J. Public Health.

[47] De Jong Gierveld J., Tilburg T.V. (2010). The De Jong Gierveld short scales for emotional and social loneliness: Tested on data from 7 countries in the UN generations and gender surveys. Eur. J. Ageing.

